# Size Increment During Surveillance in Papillary Thyroid Cancer: Evidence Synthesis and Dose–Response Meta‐Analysis

**DOI:** 10.1002/cnr2.70183

**Published:** 2025-05-08

**Authors:** Mohamed Badie Ahmed, Emad Naem, Harman Saman, Abeer Alsherawi, Asma Syed, Noora Al‐Abdulla, Latifa Alkaabi, Mahmoud A. Zirie, Suhail A. Doi

**Affiliations:** ^1^ Department of Population Medicine, College of Medicine, QU Health Qatar University Doha Qatar; ^2^ Department of Plastic Surgery Hamad General Hospital, Hamad Medical Corporation Doha Qatar; ^3^ Endocrinology Department Hamad General Hospital, Hamad Medical Corporation Doha Qatar; ^4^ Department of Medicine Hazm Mebaireek Hospital, Hamad Medical Corporation Doha Qatar; ^5^ Kent Oncology Center Kent UK

**Keywords:** active surveillance, dose–response meta‐analysis, papillary thyroid cancer, tumor progression

## Abstract

**Introduction:**

Differentiated thyroid cancer has become a prevalent malignant tumor, particularly papillary thyroid carcinoma (PTC), constituting over 90% of cases. Most PTC lesions are asymptomatic and remain subclinical throughout life, and active surveillance is now being used to monitor the progression of these lesions and intervene when appropriate. This evidence synthesis aims to assess PTC progression in size over time during active surveillance in terms of PTC tumor size progression to or beyond 3 mm during the surveillance period.

**Methods:**

A dose–response meta‐analysis was conducted using the robust error meta‐regression method, using time as the “dose” and cumulative progression (%) was assessed. A comprehensive literature review was done using PubMed/MEDLINE and EMBASE from inception till August 2024.

**Results:**

A total of 21 studies from 7 different countries, including 14 648 participants, were included. Incident progression at 2 years of follow‐up was 1%, and this increased linearly to 12% at 20 years of follow‐up.

**Conclusion:**

It was concluded that progression over the threshold increases linearly over time at approximately less than 1% per year. Therefore, size progression beyond the threshold occurs in a minority and in less than one eighth of subjects by two decades of follow‐up.

**Abbreviations**
DRMA: dose response metaanalysis
DTC: differentiated thyroid cancer
MASTER: Methodological Standards for Epidemiological Research
PTC: papillary thyroid cancer
US: ultrasound

## 1 Introduction

Differentiated Thyroid Cancer (DTC) has become one of the most frequent malignant tumors in recent years, with an increase in incidence of 3‐ to 15‐fold over the last two decades [1, 2]. The most common type of thyroid cancer is papillary thyroid carcinoma (PTC), which accounts for over 90% of all thyroid cancers. A dramatic increase in incidence has been observed in several countries, such as the USA, with a 2.9‐fold increase over the last 35 years [3, 4]. Moreover, similar observations of thyroid cancer incidence increase were observed in South Korea (especial when screening was initiated) and Japan [5, 6, 7]. However, the majority of this increase was due to a higher incidence of small PTC lesions ≤ 1 cm; other thyroid cancer types demonstrated a stable incidence rate [8]. Even though the incidence is increasing, the mortality rate has remained steady [9]. The evolution of medical treatment for DTC over time cannot justify this consistent mortality rate. As a result, it has been concluded that the higher incidence is due to DTC overdetection (a severe form of length‐time bias) rather than increased carcinogenesis over time. This has been supported by the results of autopsy studies over time [9, 10, 11].

The high prevalence of diagnosis has been matched by a high rate of thyroidectomy, which has influenced the health‐care system's expense [12]. The majority of the newly diagnosed PTC lesions are subclinical and might stay asymptomatic for the rest of the patients' lives without surgical intervention [3]. Thus, unnecessary procedures might expose the patient to anesthesia and surgical complications (hypoparathyroidism and paralysis of the recurrent or superior laryngeal nerves) without affecting the patient's prognosis [13]. These observations raised a serious question about the necessity of treatment for asymptomatic small PTCs. As early as 1993, Miyauchi conducted an observational study for low‐risk PTC without surgical intervention at Kuma Hospital; and the initial results were promising [14]. Following that, several countries began to implement the active surveillance strategy, and eventually, this entered the latest American Thyroid Association (ATA) management guideline as an alternative plan for low‐risk thyroid cancers in lieu of surgical management [15]. Although several active surveillance studies have been conducted, many variations were found among them in terms of tumor size inclusion, follow‐up period, and tumor progression rate. Uncertainty, therefore, remains regarding the exact PTC lesion progression rate during active surveillance management. In this dose–response meta‐analysis (DRMA), we now assess incident progression rates of PTC over the follow‐up periods in several studies, as knowledge of the exact progression rate will give us an idea about what to expect during active surveillance and also guide departure from what is the norm.

## 2 Methods

### 2.1 Search Strategy

Following the recommendations of the Preferred Reporting Items for Systematic Reviews and Meta‐Analyses (PRISMA) statement, a literature search strategy was developed using medical subject headings and text key words related to active surveillance in papillary thyroid cancer [16]. A comprehensive literature review was done using PubMed/MEDLINE and EMBASE from inception to August 2024. Language search was limited to English language only. Keywords used were ((Watchful Waiting) OR “Watchful Waiting”[Mesh] OR “Active surveillance”) AND thyroid AND papillary. In addition, a similar articles search and relevant references were reviewed [17].

### 2.2 Study Selection Criteria

Articles that assessed the progression of PTC (< 1.5 cm) under active surveillance in the English language and that had a cohort study design were included. All other publication types, including reviews, case reports, commentaries, letters to the editor, and preprints, were excluded. Papers assessing progression after surgery with insufficient information about patients' follow‐up or tumor progression parameters, preliminary data, conference presentations, and abstract publication were excluded. The selection of all articles was done independently by two reviewers, with disagreements resolved by including a third independent reviewer.

### 2.3 Screening and Data Extraction

Articles retrieved from the literature review passed through different screening stages. Initially, articles were screened by title, followed by abstract, then finally by the full text. After that, a similar articles search was conducted for the selected articles, and the first 25 articles for each included paper were screened. Moreover, relevant citation references were reviewed for all the selected articles. After identifying and finalizing the selected articles, one investigator was responsible for data extraction from the selected articles. The following information was collected from the selected articles: author name, publication date, study location, sample size, age, gender, number of patients with tumor increase in size, duration of follow‐up, and inclusion tumor size cut‐off. The tumor size progression cut‐off was ≥ 3 mm in all articles.

### 2.4 Quality Assessment

Methodological quality (mQ) assessment was done using the Methodological Standards for Epidemiological Research (MASTER) scale developed by Stone et al. [18]. The scale consists of five main standards that contain 36 safeguards that cover potential areas of bias in each article. Each article has a count of implemented safeguards out of 36. For ranking, each study the score is divided by the highest score among the included studies, and this rank has a possible range between 0 and 1 (the article with highest number of safeguards) [19].

### 2.5 Statistical Analysis

Descriptive statistics were used to summarize the variables in the selected studies. The effect size of interest was the progression proportion over a specific follow‐up period. This was modeled as a cumulative incidence difference in the DRMA. The latter was conducted as a 1‐step procedure with follow‐up duration (as the “dose”) along with cumulative progression incidence as the outcome using a linear robust error meta‐regression model [20]. A linear model was used because nonlinearity was not observed. In addition, included studies were divided into two groups based on the tumor size inclusion cut‐off. The first group included studies with a ≤ 1 cm cut‐off, and the other group included higher (≤ 1.1 to ≤ 1.5 cm) cut‐offs. DRMA was conducted for each group separately as a sensitivity analysis. Stata MP 15 (StataCorp, College Station, TX) was used for the analysis utilizing the robust error meta‐regression (*remr*) module in Stata [20, 21]. The confidence level was set at 95%.

### 2.6 Institutional Review Board (IRB) Approval

IRB approval was not required for this project as it is a systematic review and DRMA paper, and the data published here are based entirely on publicly available data that have been previously published in the literature.

## 3 Results

**FIGURE 1:**
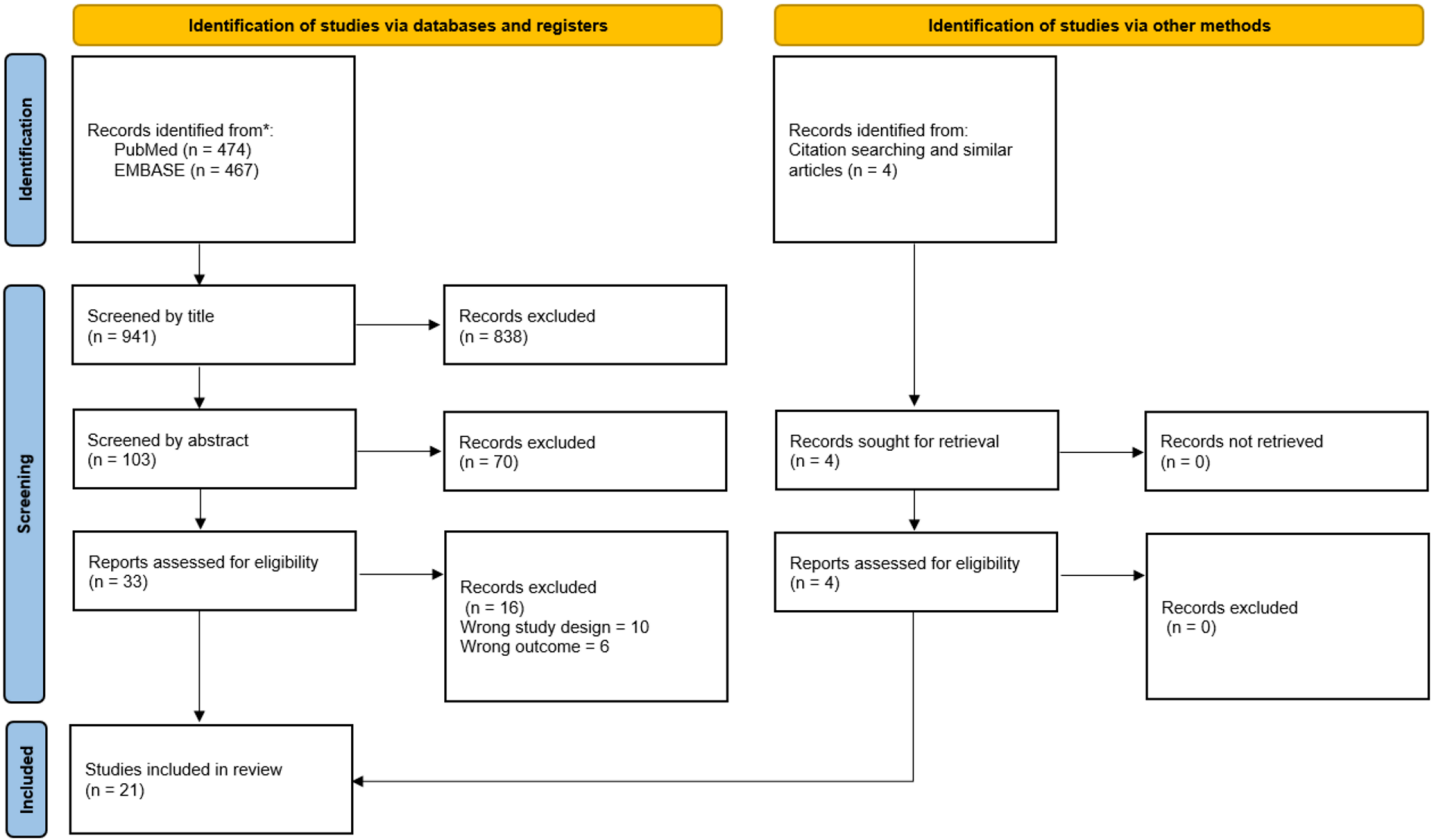
Preferred Reporting Items for Systematic reviews and Meta‐Analyses (PRISMA) flowchart of the Systematic Review and Dose–Response Meta‐Analysis showing the study selection process.

A total of 21 studies including 14 648 patients were enrolled in the DRMA [5, 6, 7, 22, 23, 24, 25, 26, 27, 28, 29, 30, 31, 32, 33, 34, 35, 36, 37, 38, 39]. Figure 1 shows the flowchart of the study selection process. Studies were conducted in seven different countries (Argentina, Brazil, Colombia, Italy, Japan, Korea and the USA). The mean age across all studies was 50.1 (Standard Deviation (SD) 10.8) years. The majority of the studies used a lesion size inclusion cutoff of ≤ 1 cm (13 studies) and the cutoffs were ≤ 1.1 to ≤ 1.5 cm in eight studies. The median follow‐up duration across all studies was 4.5 years (Interqurtile Range (IQR) 2.5, 7.3). The female percentage median across studies was 80.7 (IQR 76.8, 87.6). One study [28] included two datasets; thus, the total datasets included were 22. Another study [5] reported the number of lesions and their progression over time only; thus, the number of lesions was used in lieu of the patients' number in the analysis. The demographic and clinical characteristics of the included studies, along with mQ assessment (including study safeguard counts and ranks) are reported in Table 1. The MASTER scale ranges from 0.78 to 1 (highest methodological quality). Overall, the included studies have high scores indicating valid and reliable outcomes. The DRMA summary is shown in Table 2.

**TABLE 1:** Demographic and clinical characteristics of the included studies.

Study	Country	No. pt	Age (years)	Gender (F)	No. pt. with tumor increase in size > 3 mm	Duration of follow up	Size included	MASTER scale	Study design
Nagaoka 2021 [22]	Japan	571	53.1 ± 12.7	495 (87%)	At 5 years (4.3%), 10 (12.1%), 15 (20%), 20 (22.8%)	7.6 ± 5.0 years	≤ 1 cm	0.86	Retrospective
Campopiano 2021 [23]	Italy	109	44.2 ± 14.2	79 (72%)	2 (1.8%)	31 ± 18 months	< 1.3 cm	1	Prospective
Molinaro 2020 [24]	Italy	93	44 ± 15	72 (77%)	2 (2.2%)	19 (6–54) months	< 1.3 cm	0.93	Prospective
Sanabria 2020 [25]	Colombia	102	50.6 ± 16	85 (83%)	11 (10.8%)	13.9 (0.2–112) months	< 1.5 cm	0.89	Prospective
Miyauchi 2019 [26]	Japan	146	56 (24–79)	130 (89%)	0 (0%)	10.5 (1.57–13.5) years	≤ 1 cm	0.96	Retrospective
Rosario 2019 [27]	Brazil	77	52 (23–81)	61 (79%)	1 (1.3%)	30 months	≤ 1.2 cm	0.93	Prospective
Sakai 2019 [28]	Japan	360	53.9 ± 12.0	319 (89%)	29 (8%)	7.3 (0.5–25) years	≤ 1 cm	0.93	Retrospective
Sakai 2019 [28]	Japan	61	54.4 ± 10.7	47 (77%)	4 (7%)	7.9 (1–17) years	< 1.3 cm	0.93	Retrospective
Oh 2019 [29]	Korea	273	51.1 (IQR 42.2–61.0)	207 (76%)	12 (4.4%)	42 (IQR 28.6–60.8) months	≤ 1 cm	0.93	Retrospective
Sanabria 2018 [30]	Colombia	57	51.9 ± 14.5	48 (84%)	2 (3.5%)	13.3 (0–54) months	< 1.5 cm	0.86	Prospective
Oh 2018 [31]	Korea	370	51.1 ± 11.7	284 (77%)	13 (3.5%)	32.5 (IQR, 21.5–47.6) months	≤ 1 cm	0.93	Retrospective
Tuttle 2017 [32]	USA	291	52 ± 15	219 (75%)	11 (3.8%)	25 (6–166) months	< 1.5 cm	0.96	Prospective
Kwon 2017 [7]	Korea	192	51.3 (IQR 42.9–59.5)	145 (76%)	27 (14%)	30.1 (IQR 21.4–43.7) months	≤ 1 cm	1	Retrospective
Oda 2016 [33]	Japan	1179	57 (15–88)	1037 (88%)	27 (2.3%)	47 (12–116) months	≤ 1 cm	1	Prospective
Ito 2014 [6]	Japan	1235	≥ 60 (40%)	1111 (90%)	58 (4.6%)	60 (18–227) months	≤ 1 cm	0.93	Retrospective
Sugitani 2010 [5]	Japan	230	54 (23–84)	204 (89%)	22 of 300 lesions (7%)	Mean 5 years	< 1.1 cm	0.96	Prospective
Kim 2018 [34]	Korea	126	51 (IQR 44–58)	100 (79%)	7 (5.6%)	26 (IQR 17–37) months	≤ 1 cm	0.96	Retrospective
Smulever 2019 [35]	Argentina	34	Median 41.5	29 (85%)	6 (17.6%)	48.8 ± 29.6 months	≤ 1.5 cm	0.93	Mixed (Prospective & Retrospective)
Miyauchi 2023 [36]	Japan	3222	57.0 (20.0–92.0)	2824 (88%)	124 (3.8%)	7.3 years [1.0–29.3 years]	< 1 cm	0.78	Retrospective
Ito 2023 [37]	Japan	2705	58 (20–92)	2352 (87%)	At 5 years (3.0%), 10 years (5.5%), 15 years (6.3%)	5.5 years (1.0–15.7)	< 1 cm	0.81	Retrospective
Yamamoto 2023 [38]	Japan	2509	57.32–13.32	1901 (76%)	86 (3.43%)	6.57 years (1.02–17.6)	< 1 cm	0.81	Retrospective
Lee 2022 [39]	Korea	706	49.3 ± 11.8	578 (62.9%)	41 (5.8%)	41.4 ± 16 months	≤ 1 cm	0.83	Prospective

Abbreviations: IQR, Interquartile range; MASTER, Methodological Standards for Epidemiological Research; no., number; pt., patients.

**TABLE 2:** Descriptive results of the studies included in the dose–response meta‐analysis.

Variables	Sub‐groups	Value
Articles included		21
Country	Argentina	1 (5%)
	Brazil	1 (5%)
	Colombia	2 (10%)
	Italy	2 (10%)
	Japan	9 (43%)
	Korea	5 (24%)
	USA	1 (5%)
Size inclusion	< 1.1 cm	1 (4.5%)
	< 1.3 cm	3 (13.6%)
	< 1.5 cm	3 (13.6%)
	≤ 1 cm	13 (59%)
	≤ 1.2 cm	1 (4.5%)
	≤ 1.5 cm	1 (4.5%)
Age, mean (SD)		50.1 (10.8)

Abbreviation: SD, Standard Deviation.

**FIGURE 2:**
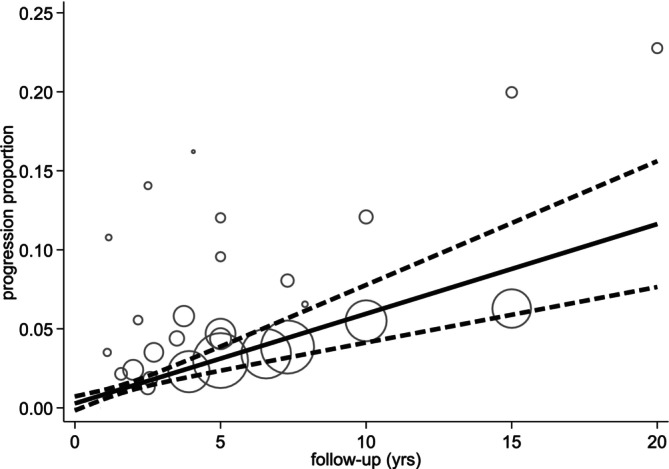
Dose–response meta‐analysis results of the 21 studies.

Results of the DRMA conducted on the 21 studies to assess the progression of PTC over a specific follow‐up duration are reported in Table 3 and Figure 2. At 2 years of follow‐up, the papillary thyroid cancer progression proportion was 1% (95% CI 1%–2%) while at 5 years, the progression proportion was 3% (95% CI 2%–4%). The longest follow‐up duration was 20 years, with a progression proportion of 12% (95% CI 8%–16%).

**TABLE 3:** Dose–response meta‐analysis results for the 21 studies.

Follow‐up (years)	Progression	(95% CI)
2	0.01	(0.01–0.02)
3.5	0.02	(0.02–0.03)
5	0.03	(0.02–0.04)
10	0.06	(0.04–0.08)
15	0.09	(0.06–0.12)
20	0.12	(0.08–0.16)

Abbreviation: CI, Confidence Interval.

**FIGURE 3:**
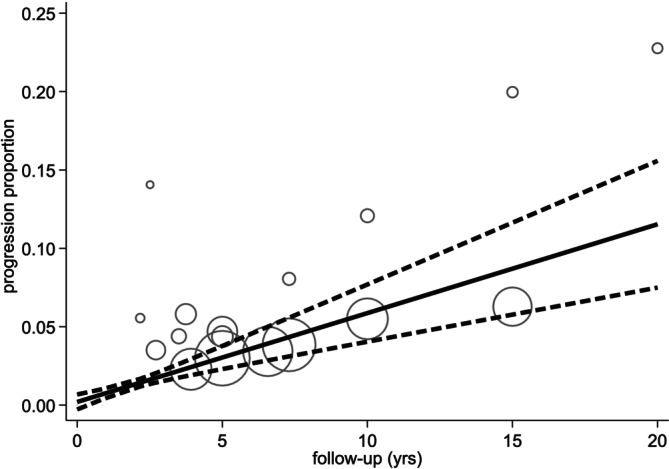
Dose–response meta‐analysis results for group 1 (≤ 1 cm).

**FIGURE 4:**
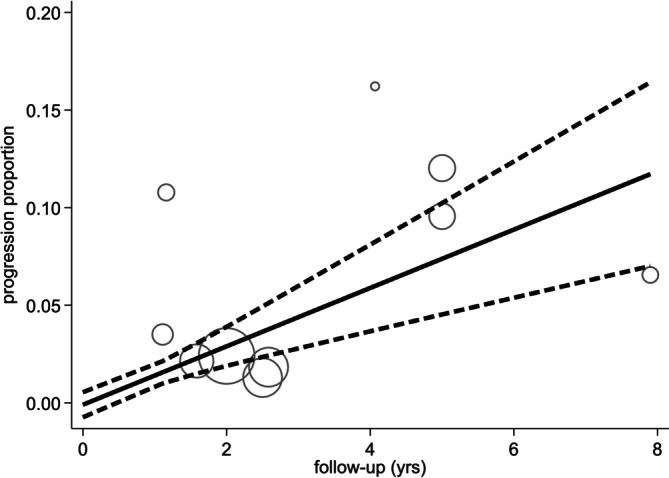
Dose–response meta‐analysis results for group 2 (> 1 cm).

A sensitivity analysis by inclusion size group (≤ 1 cm and ≤ 1.1 to 1.5 cm) to assess whether including larger tumors would affect the progression rate demonstrated a marginal increase in progression proportion in the second group (≤ 1.1 to 1.5 cm). At 5 years, the progression proportion in the first group (≤ 1 cm) was 3% (95% CI 2%–4%), while in the second group (≤ 1.1 to 1.5 cm) was 7% (95% CI 5%–10%). The dose–response relationship is depicted in Figures 3 and 4.

## 4 Discussion

This meta‐analysis represents the first DRMA that attempted to assess PTC tumor size cumulative progression over a specific follow‐up time, the threshold for progression being ≥ 3 mm, which is the smallest lesion clearly detectable by ultrasound. In general, modern ultrasound machines can detect thyroid nodules as small as 2–3 mm (mm) in diameter. However, the sensitivity of detection may vary, and nodules smaller than this size can sometimes be missed [40]. Important factors such as operators skills and experience and quality of the ultrasound equipment affect the detection rate of small nodules. Nonetheless, in their study, Ito et al. showed that only 6.7% of Papillary MicroCarcinoma of the Thyroid (PMCT) enlarged by 3.0 mm or more in diameter during 5 years of follow‐up, and nodal metastases became detectable in 1.7% of patients overall [40]. The results suggest that waiting longer will increase cumulative attainment of the progression threshold, which is a function of time and estimated to increase by around less than 1% for each year of follow‐up. In the sensitivity analysis, there was a slightly increased progression proportion when comparing the group with higher tumor size inclusion cut‐offs when compared with the other group (≤ 1 cm). These progression proportions are low enough to support the active surveillance approach and validate the low progression rate hypothesis for small size PTC lesions (< 1 cm). A tumor size progression of ≥ 3 mm is expected to be present in around 12% of subjects after 20 years follow‐up duration. This linear progression suggests that less than one eighth progress at two decades and is thus very low.

PTC is characterized by specific molecular changes that contribute to its development and progression. Understanding these molecular changes in PTC is crucial for developing strategies to predict aggressiveness that can be incorporated into AS programs as well as targeted therapies [41, 42]. These changes often involve mutations and alterations in key genes and signaling pathways. For example, the BRAF V600E mutation is one of the most prevalent molecular alterations in PTC. This mutation occurs in the BRAF gene and leads to the constitutive activation of the MAPK signaling pathway. This aberrant signaling promotes cell growth and proliferation [43, 44, 45]. BRAF V600E has emerged as a promising prognostic factor for PTC, but its clinical value as a prognostic tool remains inconclusive [46, 47, 48]. Another common genetic alteration in papillary thyroid cancer is the rearrangement of the RET gene, leading to the formation of RET/PTC fusion genes. These fusion proteins activate downstream signaling pathways, such as MAPK and PI3K/AKT, promoting uncontrolled cell growth [49, 50, 51]. Another important oncogenic change is the RAS mutation. In PTC, RAS mutations are detected in approximately 10%–20% of cases [43, 52, 53]. RAS genes, including HRAS, NRAS, and KRAS, encode small GTPases that regulate critical cellular signaling pathways involved in cell proliferation, survival, and differentiation.

These mutations are more prevalent in follicular variant PTC (FVPTC) and in older patients. RAS‐mutated PTCs often exhibit distinct clinical features, such as larger tumor size, encapsulation, and less lymph node metastasis compared to other PTC subtypes [42, 54, 55, 56]. RAS mutations in PTC result in the activation of the mitogen‐activated protein kinase (MAPK) signaling pathway. This causes overexpression of genes involved in cell cycle progression and growth, promoting tumor development. Furthermore, RAS mutations can cause resistance to targeted therapies, an important clinical consideration when choosing treatment [56, 57]. Although less frequent, mutations in the TP53 tumor suppressor gene have been observed in aggressive forms of PTC. TP53 mutations disrupt cell cycle regulation and DNA repair mechanisms [58, 59, 60]. Mutations in the TERT (telomerase reverse transcriptase) promoter are associated with increased telomerase activity, allowing cancer cells to maintain their telomeres and avoid senescence or apoptosis [61]. TERT promoter mutations are linked with aggressive thyroid cancer cell features, disease recurrence, and increased mortality [61, 62, 63]. Loss of function mutations or deletions in the PTEN gene can occur in PTC. PTEN is a tumor suppressor gene that regulates the PI3K/AKT pathway, affecting cell growth and survival, and loss of PTEN function promotes further aggressiveness of the BRAF V600E mutation [64, 65, 66, 67]. Another important molecular alteration in PTC is the loss of Thyroid Differentiation Markers: As papillary thyroid cancer progresses, there is often a loss of expression of thyroid‐specific genes like thyroglobulin and sodium‐iodide symporter (NIS). This loss of differentiation markers is associated with a more aggressive phenotype [41, 68, 69, 70]. Molecular alterations in PTC can therefore provide valuable information about prognosis and potential therapeutic targets; to date, no molecular markers or specific criteria have been conclusively shown to help in identifying the small number of patients who might progress by 3 mm that ends their active surveillance period. The decision for surgery is mainly driven by clinical, radiological, and pathological factors. Molecular findings can complement surgical selections and planning but do not directly mandate surgical intervention [71, 72, 73, 74, 75, 76].

Having a validated prognostic scoring system in a clinical setting would be a valuable tool for predicting disease progression and can aid the decision‐making process. One commonly used scoring system is the American Thyroid Association (ATA) Risk Stratification System, which assigns risk categories based on various clinical and pathological factors [77, 78, 79]. However, the following factors are accepted as clinical and radiological predictors of disease progression and therefore their presence usually precludes active surveillance and indicates upfront surgery instead: older patients (greater than 55 years), tumor diameter > 1 cm, extrathyroidal extension of cancerous tissue spreading beyond the thyroid capsule into nearby structures, such as muscles or the trachea, metastasis of cancer to local lymph nodes or distant sites, presence of a deep‐seated nodule especially those close to the tracheoesophageal groove, and tumor multifocality (i.e., multiple tumor foci within the thyroid gland) [80]. In addition, there has been a growing body of evidence that supports the presence of BRAF V600E mutation, which should also be factored in, as it is associated with a higher risk of disease progression [79, 81, 82, 83]. Nevertheless, despite the presence of such prognostic aids in predicting PTC recurrence rates and distinguishing between low‐ and high‐risk patients, there is still no international consensus on which model is optimal [84, 85, 86].

There is also ongoing controversy regarding when to end active surveillance. Many have considered an increase of ≥ 3 mm in size to be the optimum signal for indicating surgical intervention [26, 29]. Miyauchi et al. required immediate surgical intervention for tumor increase of ≥ 3 mm or lymph node metastasis, and 4% of the subjects in the study underwent surgery due to tumor size increase [26]. On the other hand, Ito et al. and Rosario et al. did not consider tumor size progression of ≥ 3 mm as an indication for surgery but rather an increase to over 1.2 cm in size [6, 27]. Another study recommended thyroid surgery for patients with tumor size greater than 1 cm; although they also considered tumor size progression of ≥ 3 mm, lymph node metastasis, or growth toward adjacent structures as equally important, and by these criteria during the follow‐up period, 19% of the subjects underwent thyroid surgery (total thyroidectomy or lobectomy) [29]. As can be inferred from the aforementioned studies, several criteria have been proposed and implemented in the active surveillance trials. Although, huge variation was noticed in the tumor size cut‐off point that indicates surgery, ranging between > 1, > 1.2, and > 1.5 cm, there seems to be a general agreement about considering a ≥ 3 mm increment as a threshold for tumor size progression. Other articles have also added time to tumor size increments, and Smulever et al. defined surgical intervention criteria for tumor size as an increase of ≥ 3 mm from the baseline in less than 1 year or tumor size larger than 1.5 cm, and around 6% of the patients had surgery due to tumor size progression of ≥ 3 mm from the baseline [35]. A justification of the increment threshold over 1 year was studies that suggested that size progression of benign and malignant thyroid nodules differed [87] with malignant nodules growing > 2 mm per year, and this was absent in the majority of benign nodules. However, this DRMA suggests that the malignant group probably represents those lesions that have been around for longer, so they are more likely to reach the threshold over time. There are indeed studies that suggest no such difference in smaller nodules [88].

To the best of our knowledge, this DRMA is the first publication in this field that attempts to assess PTC tumor size progression proportion over a specific period of follow‐up. The outcome of this analysis validates the active surveillance management plan as an alternative to surgical intervention for small PTC lesions without clinical symptoms because we document that small nodules expand at a very slow rate, allowing enough time for intervention to be planned if expansion does occur. We can safely, therefore, continue active surveillance till such expansion is seen, and we show that this accrues at 1% per year for those followed up. We, however, cannot recommend what strategy needs to be taken after expansion is seen, as the data demonstrate that expansion in size is a continuous process, and we do not have data to show the outcomes linked to such expansion. However, although several articles were included, the majority had a follow‐up period that was short (< 5 years). This might limit the validity of the size progression estimates for longer periods of follow‐up. Moreover, the studies included were published over an extended period during which diagnostic models and clinical guidelines have evolved. These differences can introduce inconsistencies and potentially affect the final outcomes. Thus, further active surveillance studies with longer follow‐up duration are needed to be able to make the results of long follow‐up stronger.

## 5 Conclusion

In conclusion, PTC incidence has increased over the past few years, and the majority of the newly diagnosed cancers are subclinical that could be managed without surgical intervention. Several countries have begun to implement an active surveillance approach instead of thyroid surgery for small‐size PTC with very promising results. Several articles have been published but with variation in tumor size inclusion, follow‐up duration, and progression outcome. This DRMA reports that the cumulative tumor size progression proportion over the follow‐up period is a linear function of time and may not be sufficient to distinguish patients requiring surgical intervention and therefore, other prognostic variables at diagnosis need to be urgently investigated in order to have optimum selection of patients for surveillance versus immediate intervention.

## 6 Author Contributions

**Mohamed Badie Ahmed:** conceptualization (supporting), methodology (supporting), data collection (lead), data analysis (lead), writing – original draft (lead), writing – review and editing (equal). **Emad Naem:** conceptualization (supporting), writing – review and editing (equal). **Harman Saman:** writing – original draft (supporting), writing – review and editing (equal). **Abeer Alsherawi:** data collection (supporting), writing – original draft (supporting), writing – review and editing (equal). **Asma Syed:** data collection (supporting), writing – review and editing (equal). **Noora Al‐Abdulla:** data collection (supporting), writing – review and editing (equal). **Latifa Alkaabi:** data collection (supporting), writing – review and editing (equal). **Mahmoud A. Zirie:** conceptualization (lead), writing – review and editing (equal). **Suhail A. Doi:** conceptualization (lead), methodology (lead), data analysis (supporting), writing – review and editing (equal).

## Ethics Statement

This article does not contain any studies with human participants or animals performed by any of the authors.

## Conflicts of Interest

The authors declare no conflicts of interest.

## Data Availability

Data sharing not applicable to this article as no datasets were generated or analysed during the current study.
